# Speciation Begins, but Doesn't End, with the Twist of a Shell

**DOI:** 10.1371/journal.pbio.0030330

**Published:** 2005-08-09

The coil of a snail shell can be either right-handed (dextral) or left-handed (sinistral), based on whether the shell spirals out clockwise or counterclockwise when viewed from above. Most species are composed entirely of individuals that are one or the other type; in exceptional cases, populations may differ in their handedness, or chirality, but within a single population, all individuals tend to be alike. This makes sense, since the mechanics of reproduction are harder between two individuals of opposite chirality (their genitalia are also reversed), reducing the likelihood that they will successfully mate and produce offspring. Over time, therefore, the rarer type will become rarer and rarer until it goes extinct.

This poses the interesting evolutionary question of how a species of one chirality can give rise to another of opposite chirality. If the rarer types are less likely to reproduce, then how do they ever establish themselves beyond a threshold frequency? If they are able to establish themselves, then is a change in chirality—which is caused by a single gene—enough to isolate them so that they are a new species? A study in this issue by Angus Davison et al. sheds light on the complex interplay of factors that influence evolution in the snail Euhadra. Although a single gene does cause a change in chirality, and snails with different chirality are able to mate only with great difficulty, there is nevertheless almost free gene flow between them. Other factors must ultimately become involved to cause speciation.

The 22 species of Euhadra are land-dwelling natives of Japan, and include five sinistral and 17 dextral species. Using mitochondrial DNA analysis to construct a family tree, the authors showed that the sinistral species compose a distinct branch, indicating that this feature arose only once in the history of the genus. How did the first sinistral shell types arise, and why didn't they gradually evaporate from the gene pool? One possibility is “reproductive character displacement,” in which a new feature that directly affects mating, such as sinistral shell chirality, decreases the likelihood that its owner will mate with snails of other, closely related, species that live nearby. While their dextral brothers or sisters waste valuable resources in such unsuccessful interspecific pairings, the few sinistral individuals engage in fewer, but more productive, matings exclusively with their own kind, thus increasing their numbers despite the odds stacked against them. [Fig pbio-0030330-g001]

**Figure pbio-0030330-g001:**
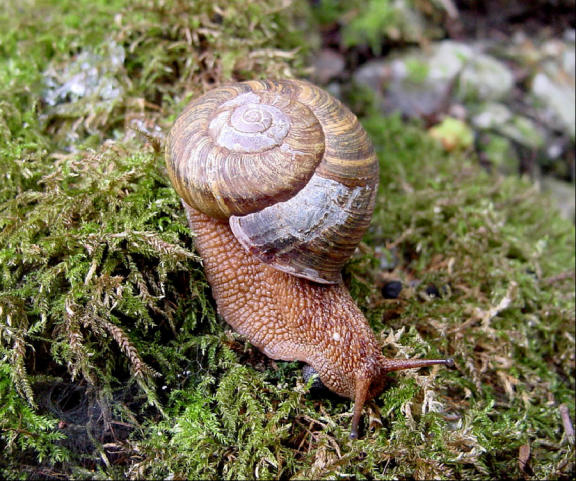
Even though snails with reversed shell spirals have trouble mating (their genitalia are also reversed), gene exchange occurs freely between two forms in a population, suggesting that speciation requires other factors

To test this hypothesis, Davison et al. constructed a model that took into account a variety of factors, including population density, the proximity of other species, and the maternal inheritance pattern of shell chirality (the direction of a snail's shell is determined so early in development that it is governed not by its own genes, but by its mother's). The surprising conclusion is that the last factor, the unusual mode of inheritance, allows for near free gene flow between the two forms within a population, even if the two forms are themselves almost unable to mate. The reason is that the offspring of a sinistral mother could itself be sinistral, even if it contains entirely dextral genes. *Its* offspring, though, might include dextral snails, because its own dextral genes determined *their* shell chirality.

Their model indicated that new chiral types are able to arise, in spite of there being fewer suitable mates, if there is reproductive character displacement. They cannot be considered new species, however, because of the gene flow between them. Reproductive character displacement can account for the speciation of sinistral Euhadra only under a complex set of conditions. Interspecific mating would need to be common among the dextral snails. High population density helps, since it allows those with the rare new form to find each other more easily. But gene flow between left and right forms would preserve the population as a single species, unless other factors, such as difference in habitat use or geographic separation, increased the isolation of the two forms. This argues against so-called “single-gene speciation,” and shows that the creation of a new species requires more than a simple twist of fate.

